# Stool-Based Proteomic Signature for the Noninvasive Classification of Crohn's Disease and Ulcerative Colitis Using Machine Learning

**DOI:** 10.14309/ctg.0000000000000925

**Published:** 2025-10-02

**Authors:** Elmira Shajari, David Gagné, Francis Bourassa, Mandy Malick, Patricia Roy, Jean-François Noël, Hugo Gagnon, Maxime Delisle, François-Michel Boisvert, Marie Brunet, Jean-François Beaulieu

**Affiliations:** 1 Laboratory of Intestinal Physiopathology, Faculty of Medicine and Health Sciences, Université de Sherbrooke, Sherbrooke, Quebec, Canada;; 2 Centre de Recherche du Centre Hospitalier Universitaire de Sherbrooke, Sherbrooke, Quebec, Canada;; 3 Department of Immunology and Cell Biology, Faculty of Medicine and Health Sciences, Université de Sherbrooke, Sherbrooke, Quebec, Canada;; 4 Allumiqs, 975 Rue Léon-Trépanier, Sherbrooke, Quebec, Canada;; 5 Department of Pediatrics, Faculty of Medicine and Health Sciences, Université de Sherbrooke, Sherbrooke, Quebec, Canada;; 6 Department of Medicine, Faculty of Medicine and Health Sciences, Université de Sherbrooke, Sherbrooke, Quebec, Canada.

**Keywords:** stool biomarkers, Crohn's disease, ulcerative colitis, inflammatory bowel disease subtyping, DIA mass spectrometry, quantitative proteomics, machine learning

## Abstract

**INTRODUCTION:**

Crohn's disease (CD) and ulcerative colitis (UC) have overlapping symptoms, but they differ in pathology and treatment. Currently, distinguishing between these diseases involves invasive procedures such as colonoscopy and histopathology. Fecal proteins, stable and in direct contact with inflammation, offer a noninvasive alternative. This study focuses on using high-throughput data-independent acquisition mass spectrometry and machine learning to develop an accurate biomarker signature from complex stool samples.

**METHODS:**

Stool samples obtained from 69 active patients were analyzed. Analysis of the stool proteome led to the identification and quantification of approximately 1,250 proteins. The samples were divided into training and testing groups. After data processing, various feature selection algorithms were applied on the training group to determine proteins that were significantly different between the CD and UC groups. In addition, 6 machine learning algorithms were evaluated to identify the best-performing classifiers.

**RESULTS:**

Sixteen proteins were selected based on several feature selection algorithms, and 6 models were trained based on them. According to the performance metrics of each algorithm on the training data set, the Naive Bayes model was selected. For performance validation, the final predictive model was applied to 16 blind prospective samples as the test data set. Notably, the model achieved an area under the curve of 0.96 on both the training and test data sets, highlighting its robustness and stability.

**DISCUSSION:**

This study demonstrates the potential of combining multiple stool protein biomarkers through high-throughput data-independent acquisition mass spectrometry and machine learning tools to develop a predictive model for efficiently distinguishing CD from UC.

## Graphical abstract

**Figure:**
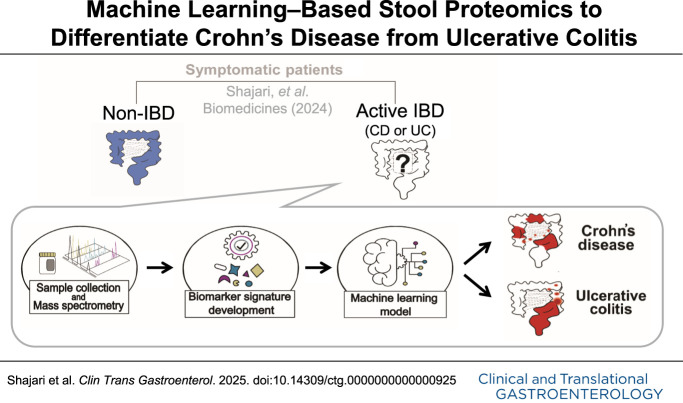

## INTRODUCTION

Crohn's disease (CD) and ulcerative colitis (UC) are the 2 most common chronic inflammatory bowel diseases (IBDs). Accurately distinguishing between these conditions would allow physicians to tailor treatment plans, including the choice of medication and surgical intervention, anticipate potential complications, and offer a more accurate prognosis, leading to improved overall management of IBD (1). The differential diagnosis of CD and UC using noninvasive methods remains inconsistent, thus still relying on invasive endoscopy and histopathology (2). However, even with these methods, diagnosis can remain challenging for gastroenterologists.

Stool biomarkers provide a valuable, noninvasive method for investigating intestinal inflammation because they are in direct contact with the affected area (3,4). Numerous studies have explored protein biomarkers for diagnosing and differentiating CD and UC; however, each has its limitations. Many studies focused on only diagnosing 1 group, either CD or UC, from a control group (5). This approach may not be practical in real-world settings because distinguishing CD from controls without considering UC is not feasible. Moreover, a microbiome proteome study developed a model that performed well on the training data set but poorly in validation, achieving an area under precision recall curve of 0.47, indicating its limited accuracy in distinguishing CD from UC (6). Other studies have relied on mucosal proteomics, which involves biopsy procedures that remain invasive (7,8). In another study, although stool metaproteomic analyses identified differentially expressed proteins, they failed to distinguish CD from UC (9). These limitations highlight the need for more effective and noninvasive approaches to distinguishing between these 2 types of IBD (10).

Recent advances in proteomics platforms hold great promise for identifying and measuring multiple targets simultaneously. In particular, Sequential Window Acquisition of All Theoretical Mass Spectra (SWATH) mass spectrometry as data-independent acquisition (DIA) offers a high-throughput approach to stool proteomic profiling (11–13). In a previous study, we demonstrated the effectiveness of this method in differentiating active IBD from non-IBD patients, leveraging machine learning for enhanced diagnostic accuracy (14).

Building on these findings, this study investigates whether distinct stool proteomic signature can differentiate CD from UC. Using SWATH/DIA proteomic profiling combined with machine learning, we identified 16 proteins that significantly improve diagnostic precision. The model's robustness was validated with independent samples, demonstrating its strength and consistency. All samples were collected under clinically compatible standard operating procedures, ensuring clinical relevance.

## METHODS

### Stool sample collection

Stool samples were obtained from the Clinical Hematology Laboratory at CIUSSS de l'Estrie—CHUS through the fecal calprotectin testing program between January 2021 and August 2023. Patients collected samples at home and delivered them to the hospital within 24 hours (2 hours at room temperature, up to 24 hours refrigerated at 4°C). Approximately 50 mg was used for calprotectin testing by ELISA, while the remainder was stored at −80°C for this study, with patient consent.

Criteria for inclusion required patients to be adults with active disease, defined by evidence of endoscopic activity, elevated fecal calprotectin levels, and relevant clinical history. Histologic activity was considered when available; however, histologic activity without clinical or endoscopic activity was not considered as active-CD (aCD) or active-UC (aUC). Samples with ambiguous diagnoses were excluded.

The study was conducted in accordance with the Declaration of Helsinki and approved by the Institutional Research Ethics Committee of the CIUSSS de l'Estrie—CHUS (protocol code 1991-17, 90-18, last date of approval 2025-08-27).

### Sample preparation for mass spectrometry analysis

Sample preparation followed the previously described protocol (14). Briefly, 100 mg of frozen stool was dissolved in lysis buffer (25 mM Tris, 1% sodium dodecyl sulfate, pH 7.5), centrifuged, and protein concentration was measured through bicinchoninic acid assay in the aqueous phase. Samples were reduced (10 mM dithiothreitol), alkylated (15 mM iodoacetamide), and quenched (10 mM dithiothreitol). Proteins were precipitated with cold acetone and methanol, digested using Trypsin/Lys-C, and peptides were purified with Strata-X SPE columns, dried at 37°C for 45 minutes, and stored at −80°C. Before liquid chromatography-tandem mass spectrometry (LC-MS/MS) analysis, peptides were resuspended in 20 μL mobile phase (0.2% formic acid, 3% dimethyl sulfoxide in water).

### Identifying and quantifying proteins and peptides in stool samples

The acquisition of SWATH LC-MS/MS data was conducted as previously described (14,15). Briefly, proteomic analysis was performed at the Allumiqs Solutions proteomics facility using an Eksigent μUHPLC system coupled with an ABSciex TripleTOF 6600 mass spectrometer in DIA mode. Chromatographic separation was achieved on a reverse-phase column, and SWATH acquisition parameters were optimized using the SWATH Variable Window Calculator (Sciex).

Spectral library generation involved processing proteins from pooled samples on a polyacrylamide gel, followed by peptide extraction. Data were acquired in data-dependent acquisition mode and searched against the human proteome database using MSFragger (FragPipe v19.1) (16). The resulting spectral library included ∼2000 proteins. Label-free quantification was performed with DIA-NN software (v1.8.1) using match between run mode, which transfers peptide identifications across runs based on accurate mass and retention time alignment to increase data completeness, applying gene-based protein inference at a 1% false discovery rate threshold. Full technical details, including LC gradient conditions and normalization strategies, are provided in the supplementary material (17).

### Statistical analysis for discovering potential biomarkers

**Figure 1.**
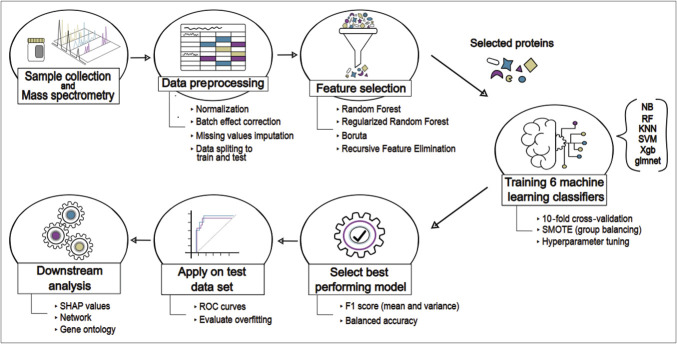
Schematic representation of the workflow for biomarker selection and developing the predictive machine learning model. KNN, k-nearest neighbors; NB, Naive Bayes; RF, Random Forest; ROC, receiver operating characteristic; SHAP, SHapley Additive exPlanations; SMOTE, Synthetic Minority Oversampling Technique; SVM, Support Vector Machine; Xgb, eXtreme Gradient Boosting.

The key steps of the methodology are summarized in Figure 1. A total of 69 stool samples were analyzed across 4 mass spectrometry batches. Eighty percent of the samples (35 aCD, 18 aUC) were used for training a predictive model, while the remaining 16 samples served as a blind test set for validation.

Data preprocessing followed previously described protocols (14) including log2 transformation, quantile normalization (preprocessCore package (18)), and batch effect correction (ComBat function, sva package (19)). Missing values were imputed using the K-nearest neighbors (KNN) method (impute package (20)). Differentially expressed proteins (DEPs) were identified using ProStar (v1.30.5) (21) through the Welch *t* test (fold change ≥1.5, *P* < 0.05, adjusted for the false discovery rate using the st.boot method).

We also used 4 distinct feature selection algorithms in R including (i) Recursive Feature Elimination and (ii) Boruta from Boruta package (22), and (iii) Random Forest (RF) and (iv) Regularized Random Forest (RRF) from RRF package (23). These algorithms were used to compute and compare the statistical significance of potential biomarkers in our data set.

### Implementation of machine learning algorithms

Six machine learning algorithms were trained for binary classification: KNN, Naive Bayes (NB), eXtreme Gradient Boosting, RF, Support Vector Machine, and Lasso and Regularized Logistic Regression (glmnet), implemented in caret package in R (24).

Models were trained using the training data set, and their performance was evaluated based on sensitivity, specificity, precision, recall, F1-score (harmonic mean of precision and recall), and balanced accuracy to distinguish aCD from aUC. The analysis was conducted using 10-fold cross-validation with 3 repeats to minimize the risk of overfitting. Hyperparameter tuning was conducted using tuneGrid, optimizing model parameters for the best performance. The Synthetic Minority Oversampling Technique was used to address class imbalance in the data set. The best-performing model was selected based on validation metrics and applied to a blind testing data set for final evaluation.

### Network and gene ontology analysis of differentiating proteins

We conducted a network analysis of the 51 differentiating proteins, assessing key metrics such as centrality and betweenness to determine their network importance. The network was built using STRING database (v12.0). In addition, we analyzed the biological processes and molecular functions of these proteins using Gene Ontology (GO) terms (25,26) to classify their roles. This approach clarifies their biological interactions and highlights their functional significance. We also retrieved the GO term using GOATOOLS (v1.2.3) (27), in Python, with the 2024-08-01 GO annotations and 2024-06-17 GO definitions. For Table 1, the smallest level term was chosen for annotation. To identify common terms within the network (Table 3), the human proteome (UniProtKB: UP000005640) or the detected proteome served as the background set, while the 51 differentially expressed proteins were used as the query set.

## RESULTS

### Patient demographics and stool sample distribution

A total of 69 stool samples were collected, comprising 46 from patients with aCD and 23 from patients with aUC. The aCD samples included a mix of ileal (55%), colonic (21%), and ileocolonic (24%) cases. The age range of participants was from 19 to 83 years, with a mean age of 49.5 years. The sex distribution was nearly equal across both groups, with 53% female and 47% male.

### Protein identification and feature selection

**Figure 2.**
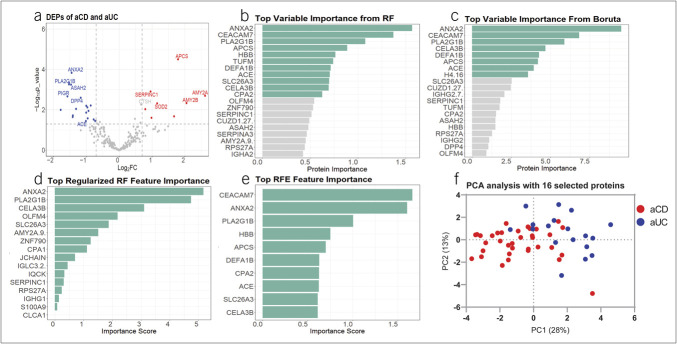
(**a**) The volcano plot displays DEPs, with blue for upregulated proteins in aCD and red for aUC, based on Log_2_FC and *P* value. (**b**–**e**) Bar plots show feature importance from different selection methods: RF (**b**), Boruta (**c**), Regularized RF (**d**), and RFE (**e**), with green bars highlighting confirmed important features. (**f**) PCA plot using 16 selected biomarkers shows a clear separation between aCD and aUC, validating their effectiveness in distinguishing the 2 conditions. aCD, active Crohn's disease; aUC, active ulcerative colitis; DEP, differentially expressed protein; Log_2_FC, Log2 fold change; PCA, principal component analysis; RF, Random Forest; RFE, Recursive Feature Elimination.

From the SWATH-MS data, we identified ∼8,000 peptides, which were mapped to ∼1,250 human proteins using gene-level inference with DIA-NN and a custom data-dependent acquisition-derived spectral library. After removing contaminants and filtering for proteins with less than 30% missing values across samples, 250 high-confidence proteins were retained for downstream analysis. After normalization, batch effect correction, and missing value imputation, we identified 51 DEPs between aCD and aUC, based on *P* value and fold change (Figure 2a). Of these, 32 were higher in aUC and 19 were higher in aCD. Table 1 lists these 51 DEPs along with their primary molecular functions and biological processes.

**Table 1.** Differentially expressed proteins between Crohn's disease and ulcerative colitis with associated gene ontology terms

Name		ID	Biological process	Molecular function
ACE^a^	Angiotensin-converting enzyme	P12821	Hormone metabolic process	Heterocyclic compound binding
AMY1A	Alpha-amylase 1A	P0DUB6	Carbohydrate metabolic process	Chloride ion binding
AMY2A^a^	Pancreatic alpha-amylase	P04746	Carbohydrate catabolic process	Chloride ion binding
AMY2B	Alpha-amylase 2B	P19961	Carbohydrate metabolic process	Metal ion binding
ANXA2^a^	Annexin A2	P07355	Response to activity	Protein binding
APCS^a^	Serum amyloid P-component	P02743	Protein folding	Protein binding
ASAH2	Neutral ceramidase	Q9NR71	Ceramide metabolic process	N-acylsphingosine amidohydrolase activity
AZU1	Azurocidin	P20160	Intracellular signal transduction	Protein binding
CEACAM7^a^	Carcinoembryonic antigen-related cell adhesion molecule 7	Q14002	Signal transduction	Protein tyrosine kinase binding
CELA3B^a^	Chymotrypsin-like elastase family member 3B	P08861	Proteolysis	Peptidase activity
CLCA1	Calcium-activated chloride channel regulator 1	A8K7I4	Monoatomic ion transmembrane transport	Metalloendopeptidase activity
CLCA4	Calcium-activated chloride channel regulator 4	Q14CN2	Monoatomic ion transmembrane transport	Metalloendopeptidase activity
CPA1^a^	Carboxypeptidase A1	P15085	Response to cadmium ion	Protein binding
CPA2^a^	Carboxypeptidase A2	P48052	Proteolysis	Carboxypeptidase activity
CUZD1	CUB and zona pellucida-like domain-containing protein 1	Q86UP6	Cell division	
DEFA1B^a^	Neutrophil defensin 1	P59665		
DMBT1	Deleted in malignant brain tumors 1 protein	Q9UGM3	Vesicle-mediated transport	Protein binding
DPP4	Dipeptidyl peptidase 4	P27487	Cell adhesion	Protein binding
FBXO3	F-box only protein 3	Q9UK99	Response to lipopolysaccharide	Protein binding
GAPDH	Glyceraldehyde-3-phosphate dehydrogenase	P04406	Microtubule cytoskeleton organization	Protein binding
H3-3B	Histone H3.3	P84243		
H4-16^a^	Histone H4.16	P62805		
HBB^a^	Hemoglobin subunit beta	P68871	Regulation of blood pressure	Peroxidase activity
IGHA1	Immunoglobulin heavy constant alpha 1	P01876	Immune response	Antigen binding
IGHA2	Immunoglobulin heavy constant alpha 2	P01877	Immune response	Antigen binding
IGHG1	Immunoglobulin heavy constant gamma 1	P01857	Adaptive immune response	Protein binding
IGHG2	Immunoglobulin heavy constant gamma 2	P01859	Adaptive immune response	Protein binding
IGLC3	Immunoglobulin lambda constant 3	P0DOY3	Adaptive immune response	Antigen binding
IQCK	IQ domain-containing protein K	Q8N0W5		
JCHAIN	Immunoglobulin J chain	P01591	Immune response	Protein-macromolecule adaptor activity
LCN2	Neutrophil gelatinase-associated lipocalin	P80188	Innate immune response	Protein binding
LYZ	Lysozyme C	P61626	Killing of cells of another organism	Lysozyme activity
MEP1A	Meprin A subunit alpha	Q16819	Epidermal growth factor receptor ligand maturation	Protein binding
OLFM4^a^	Olfactomedin-4	Q6UX06	Signal transduction	Structural molecule activity
PIGR	Polymeric immunoglobulin receptor	P01833	Immunoglobulin transcytosis in epithelial cells mediated by polymeric immunoglobulin receptor	Polymeric immunoglobulin binding
PLA2G1B^a^	Phospholipase A2	P04054	Signal transduction	Signaling receptor binding
PLG	Plasminogen	P00747	Tissue remodeling	Protein binding
PRTN3	Myeloblastin	P24158	Collagen catabolic process	Protein binding
PYHIN1	Pyrin and HIN domain-containing protein 1	Q6K0P9	Activation of innate immune response	Double-stranded DNA binding
RNF170	E3 ubiquitin-protein ligase RNF170	Q96K19	Response to activity	Protein binding
RPS27A	Ubiquitin-ribosomal protein eS31 fusion protein	P62979	Translation	Structural constituent of ribosome
S100A9	Protein S100-A9	P06702	Modulation of process of another organism	Antioxidant activity
SERPINA3	Alpha-1-antichymotrypsin	P01011	Maintenance of gastrointestinal epithelium	Protein binding
SERPINC1	Antithrombin-III	P01008	Blood coagulation	Protein binding
SLC26A3^a^	Chloride anion exchanger	P40879	Sperm capacitation	Protein binding
SOD2	Superoxide dismutase [Mn], mitochondrial	P04179	Response to activity	Superoxide dismutase activity
TUFM^a^	Elongation factor Tu, mitochondrial	P49411	Translational elongation	Protein binding
ZNF790^a^	Zinc finger protein 790	Q6PG37	Regulation of transcription by RNA polymerase II	DNA-binding transcription factor activity, RNA polymerase II-specific

a These rows are the features used in the final model.

In Figure 2b–e, the plots represent feature importance for each feature selection method including RF, Boruta, RRF, and Recursive Feature Elimination. Each algorithm uses different methods to assign a score to each protein. By comparing the results of these methods, we selected the top important features, identified by at least 1 feature selection method, resulting in 16 proteins. Notably, annexin A2 (ANXA2), group IB phospholipase A2 (PLA2G1B), and chymotrypsin-like elastase 3B consistently received top scores across all feature selection methods. Figure 2f indicates that the principal component analysis based on these 16 selected proteins allows for clear differentiation between the classes.

### Selected proteins expression levels

**Figure 3.**
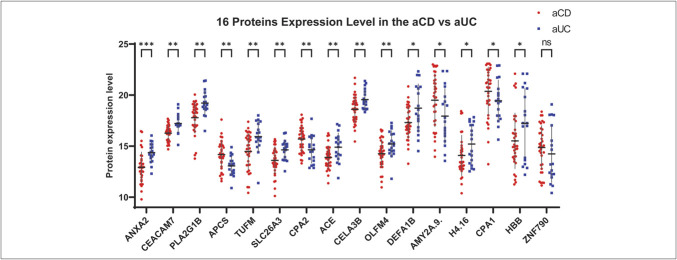
The plot shows the expression levels of 16 selected proteins in active Crohn's disease (aCD, red) and active ulcerative colitis (aUC, blue) patients. Statistical significance between the 2 groups was assessed using the Mann-Whitney test. Significant differences are marked by asterisks (***q-value < 0.001, **q-value < 0.01, *q-value < 0.05), and “ns” denotes nonsignificant differences. Horizontal lines represent the median with SD protein expression levels for each group. Despite these significant differences, there is no single threshold that can reliably distinguish between the groups, indicating partial overlap. This underscores the necessity of using machine learning for effective classification.

The expression levels of these 16 proteins in aCD and aUC samples are depicted in Figure 3. Significant differences are observed. ANXA2 showed the most significant differences, and zinc finger protein 790 exhibited no significant difference based on the Mann-Whitney *U* test. However, no single protein's expression level consistently distinguished the 2 groups because partial overlap between aCD and aUC samples is evident for each protein. This necessitates the use of machine learning for effective classification.

### Supervised machine learning model evaluation

Model performance was evaluated using average F1 scores and balanced accuracy, along with their variance across folds in Table 2 with the corresponding best-tuned hyperparameters. Since cross-validation randomly splits the data set into different training and validation sets for each fold, slight variations in training data led to fluctuations in model performance. Although eXtreme Gradient Boosting and RF reached 100% accuracy, their lower balanced accuracy and F1 scores suggest overfitting, indicating poor generalization. By contrast, NB and KNN showed high performance with lower variance, making them the best candidates for further evaluation on unseen data.

**Table 2.** Performance comparison of machine learning classifiers in the training data set

Training							
Model	Sensitivity	Specificity	Mean F1	Variance F1	Mean balanced accuracy	Variance balanced accuracy	Tuning parameters
NB	0.86	0.89	0.88	0.02	0.83	0.05	fL = 0.25, usekernel = F, adjust = 0
KNN	0.94	0.83	0.87	0.01	0.82	0.05	k = 3
GLMNet	0.86	0.83	0.86	0.01	0.79	0.06	Lambda = 0.3, alpha = 0
RF	1	1	0.85	0.01	0.78	0.07	mtry = 5
XGBoost	1	1	0.84	0.01	0.76	0.07	max_depth = 3, gamma = 0.1
SVM	0.91	1	0.84	0.02	0.74	0.09	degree = 1, scale = 0.1, C = 1

The F1 score reflects the balance between precision and recall, while the balanced accuracy accounts for imbalanced data sets by averaging the true positive rate and true negative rate. The table also includes the variance of the metrics across the cross-validation folds, indicating the stability of each classifier's performance.

KNN, k-nearest neighbors; NB, Naive Bayes; RF, Random Forest; SVM, Support Vector Machine; XGBoost, eXtreme Gradient Boosting.

### Performance on test data set

**Figure 4.**
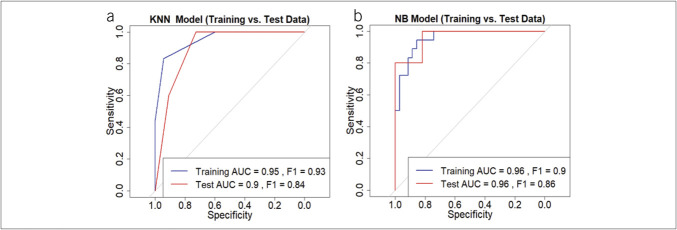
Performance comparison of K-nearest neighbors (KNN) and Naive Bayes (NB) models, selected based on training results, on the test data set. (**a**) KNN model receiver operating characteristic (ROC) curve comparison for training and test data sets shows overfitting, with a training area under the curve (AUC) of 0.95 and test AUC of 0.90 and the F1 score dropping from 0.93 (training) to 0.84 (test). (**b**) NB model ROC curve shows consistent performance with both training and test AUC of 0.96 with NB prediction of 86% sensitivity, 89% specificity, and 87% accuracy for the training data set and 82% sensitivity, 80% specificity, and 81% accuracy for the test data set.

The performance of NB and KNN on the 16-sample test data set is shown in receiver operating characteristic curves (Figure 4a, b). KNN had a training area under the curve (AUC) of 0.95 and a test AUC of 0.90, with a drop in F1 score from 0.93 to 0.84, suggesting some overfitting. NB, however, showed consistent performance (training AUC = 0.96 [95% confidence interval (CI) 0.91–1.00], test AUC = 0.96 [95% CI 0.88–1.00]), with no signs of overfitting. For NB, sensitivity, specificity, and accuracy were 0.86, 0.89, and 0.87, respectively (95% CI 74.66%–94.52%), reinforcing the model's robustness in training. In testing, these metrics remained strong at 0.82, 0.80, and 0.81 (95% CI 0.55–0.96), further supporting its generalizability. Based on these results, NB was selected as the final classifier for distinguishing aCD from aUC.

### SHAP value

**Figure 5.**
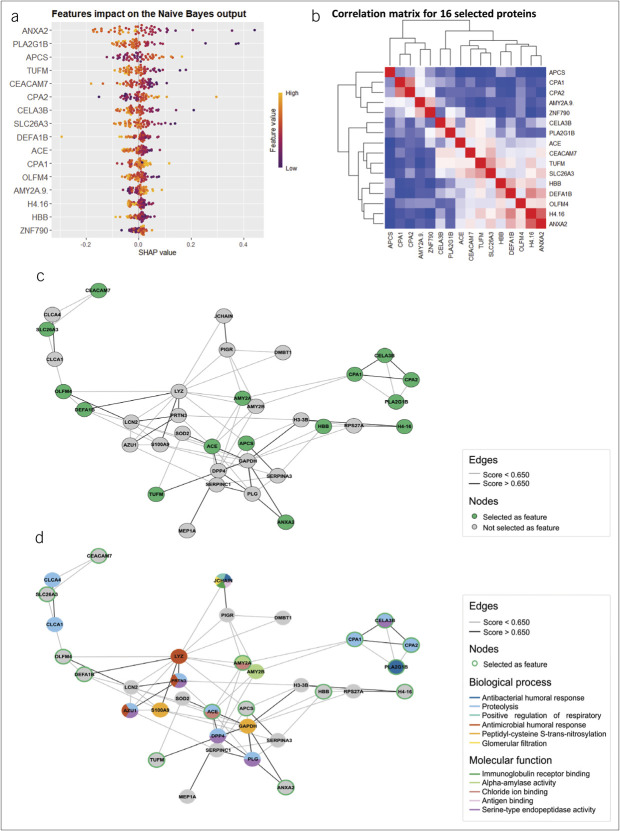
(**a**) SHAP analysis shows the impact of each protein in the Naive Bayes model using 16 selected features. Features are ranked by importance, with each dot representing an individual observation. Colors indicate feature values (yellow for high, purple for low). (**b**) Pearson correlation clustering confirms complementary effects among medium-impact features. (**c**) An interconnected network of 51 differentially expressed proteins, with selected proteins highlighted in green. Several proteins were not found in the STRING database (CUZD1, IGHA1, IGHA2, IGHG1, IGHG2, and IGLC3), and others were found but showed no connections (AMY1A, ASAH2, FBXO3, IQCK, PYHIN1, RNF170, and ZNF790). (**d**) Expanded network analysis links DEPs to biological processes and molecular functions, with PLA2G1B, ACE, CPA1-2, and CELA3B playing roles in immune response and proteolysis.

To assess the contribution of each feature (protein) in the NB model, we used SHapley Additive exPlanations (SHAP) values (Figure 5a). Features are ranked by impact, with colors indicating values for each observation. ANXA2 and PLA2G1B were the most influential, with low values strongly predicting aCD. Similarly, low amyloid P component, serum (APCS, also known as SAP) values were highly associated with aUC. Other features had moderate effects, suggesting the model captures correlations, further explored in the Pearson correlation plot (Figure 5b).

### Network analysis

We analyzed the 51 DEPs to determine their connectivity (Figure 5c). Fifteen proteins formed an interconnected network, apart from APCS, angiotensin-converting enzyme (ACE), and Amylase, which act as central hubs, mostly positioned at the periphery rather than the core, indicating although these proteins are part of the broader biological network, they might not serve as central hubs but still play important roles in specific processes or pathways. Supplementary Table S1 (Supplementary Digital Content 1, http://links.lww.com/CTG/B394) highlights proteins such as ACE, pancreatic alpha-amylase, carboxypeptidase A1, and APCS with high centrality and betweenness, suggesting their importance within the network. Figure 5d expands the analysis to include biological processes and molecular functions, with selected proteins marked in green. Key biomarkers (PLA2G1B, ACE, carboxypeptidase A1-2, and chymotrypsin-like elastase 3B) are linked to antibacterial humoral response and proteolysis, suggesting their potential involvement in immune response and metabolism.

### Common gene ontology terms across DEPs

We retrieved GO terms associated with the 51 differentially expressed proteins to identify common biological processes (Table 3). The 16 selected proteins were highlighted among contributing proteins. Key processes included antibacterial immune response and proteolysis, involving 5 proteins.

**Table 3.** Biological processes associated with DEPs in CD and UC, with selected proteins highlighted

GO term	GO name	DEP	Corrected *P* value (WP)^a^	Corrected *P* value (DP)^b^
GO:0019731	Antibacterial humoral response	IGHA1, IGHA2, JCHAIN, IGHG2, IGHG1, **PLA2G1B**	1.34E-05	0.4401
GO:0006508	Proteolysis	FBXO3, PRTN3, CLCA4, PLG, **ACE**, **CPA1**, **CPA2**, **CELA3B**, CLCA1, AZU1, DPP4	2.47E-05	0.4705
GO:0060267	Positive regulation of respiratory burst	IGHA1, IGHA2, JCHAIN	0.0012	0.331
GO:0006958	Complement activation, classical pathway	IGHG2, IGHA1, IGHA2, IGHG1	0.0042	—
GO:0019730	Antimicrobial humoral response	LYZ, PRTN3, AZU1	0.0047	0.6207
GO:0035606	Peptidyl-cysteine S-trans-nitrosylation	S100A9, GAPDH	0.0089	0.8493
GO:0050853	B cell receptor signaling pathway	IGHG2, IGHA1, IGHA2, IGHG1	0.0104	0.8493
GO:0003094	Glomerular filtration	IGHA1, IGHA2, JCHAIN	0.0151	0.4401
GO:0034987	Immunoglobulin receptor binding	IGHA1, IGHA2, JCHAIN, IGHG2, IGHG1	1.32E-06	0.02
GO:0004556	Alpha-amylase activity	AMY1A, AMY2A, AMY2B	0.0002	0.052
GO:0031404	Chloride ion binding	AMY1A, AMY2A, **ACE**	0.0049	0.1345
GO:0003823	Antigen binding	IGHA1, IGHA2, JCHAIN, IGHG2, IGHG1	0.0076	—
GO:0004252	Serine-type endopeptidase activity	PRTN3, PLG, CELA3B, AZU1, DPP4	0.0302	—

*P* value was corrected with the Benjamini-Hochberg procedure.

CD, Crohn's disease; DEP, differentially expressed protein; GO, Gene Ontology; UC, ulcerative colitis.

a Background set WP: whole proteome (i.e., biology-wide null hypothesis).

b Background set DP: detected proteome (i.e., assay-based null hypothesis).

The prominence of the antibacterial immune response suggests that the immune system's defense against bacteria differs between CD and UC, likely due to how each disease interacts with the gut microbiota. Proteolysis, crucial for immune regulation and inflammation control, seems more active in CD, where deeper tissue damage occurs, compared with the surface-level inflammation seen in UC. These findings highlight the distinct disease mechanisms.

## DISCUSSION

In this study, we explored the potential of using SWATH-DIA proteomic profiling of stool samples to distinguish between CD and UC active patients. Our process included 4 main steps: acquiring and processing data, selecting complementary protein biomarkers, training, and optimizing a machine learning model with 53 retrospective samples and validating the model's performance with 16 prospective samples. Remarkably, our model achieved an AUC of 0.96 on both training and test data sets, demonstrating its robustness and stability. This success highlights our ability to effectively process data from 4 different batches, select the best markers, and develop a reliable model based on them for distinguishing CD from UC.

To our knowledge, no stool-based test has achieved this level of distinction between CD and UC; prior work in this area has largely focused on IBD vs non-IBD or relied on different sample types. Fecal calprotectin, for example, is widely used to detect intestinal inflammation but cannot differentiate between the 2 major subtypes of IBD. Fecal calprotectin is a common stool marker of intestinal inflammation but does not distinguish CD from UC. Serologic adjuncts (anti-saccharomyces cerevisiae antibodies/perinuclear anti-neutrophil cytoplasmic antibodies) provide only moderate subtype discrimination (28). Recent reviews published in 2022 and 2023 have summarized the landscape of noninvasive IBD biomarkers, categorizing them into serum proteins, serological antibodies, and fecal proteins, and concluded that current options remain suboptimal for CD vs UC classification and are not ready for clinical use (10,29,30). For example, 1 study using a panel of 7 serological markers achieved an AUC of only 0.82, and a machine learning-based microbiome analysis reported similar microbiome profiles in CD and UC, limiting its discriminative utility (31–33). By contrast, our multiprotein stool-based signature demonstrated high accuracy and robustness in this proof-of-concept study, suggesting its potential as a future tool for precision IBD subtyping.

ANXA2, carcinoembryonic antigen-related cell adhesion molecule 7 (CEACAM7), and PLA2G1B consistently ranked among the top features across most selection methods. In addition, the Mann-Whitney *U* test conducted on 16 selected proteins confirmed that ANXA2, CEACAM7, and PLA2G1B exhibited the most significant difference. Furthermore, protein APCS and elongation factor Tu, mitochondrial (TUFM) also emerged as the next top contributors in this analysis. SHAP value analysis reinforced this finding showing that these proteins were among the most impactful features influencing our final model's decision-making. These findings are supported by previous studies that have explored the roles of these proteins in IBD, with a specific focus on UC and CD, as mentioned below.

ANXA2 which showed higher expression in UC compared with CD in this study, is an important member of the annexin family with a role in cancer progression and inflammation (34). Zhang et al (35) found ANXA2 mRNA expression significantly higher in UC than in CD and healthy controls, correlating with UC severity and histopathological grade. Furthermore, a review by the Tanida group proposed ANXA2 as a potential molecular target for UC treatment (36). These studies highlighted the importance of ANXA2 in differentiating patients with UC from CD.

PLA2G1B, a key pancreatic enzyme, was higher in UC than CD and is involved in fat metabolism, immune function, and gastrointestinal inflammation (37,38). Haller et al (39) found PLA2G1B contributes to IBD because its inactivation protected mice from DSS-induced colitis, suggesting it as a therapeutic target. Although PLA2 group 2 is linked to Crohn's inflammation (40), research on PLA2 group 1 in UC vs CD differentiation remains limited.

CEACAM7, a member of the CEACAM family (41), is specifically expressed in pancreatic and colorectal epithelial cells, particularly on the apical surface of intestinal epithelial cells (IECs). It plays an essential role in normal cellular differentiation and proliferation (41). A microarray analysis of IBD colon biopsies showed CEACAM7 levels significantly decreased in both UC and CD compared with controls, with no difference between the 2 diseases (8). Our analysis found higher CEACAM7 levels in UC than CD, suggesting a more active role in UC pathology, possibly due to differences in mucosal inflammation or epithelial damage.

APCS, a top-ranking biomarker, is higher in CD than UC and regulates inflammation by interacting with the complement system. Research by Wang et al underscores APCS's role as a versatile immunomodulator (42). Torres et al (43) found that APCS, combined with other serum markers, improves CD vs healthy differentiation. Wang et al (44) confirmed higher APCS expression in CD tissue than UC, highlighting its role in CD-UC differentiation.

Using only the top 5 proteins (ANXA2, CEACAM7, PLA2G1B, APCS, and TUFM) yielded slightly lower but still good performance, yet with a larger train-test gap than the 16-protein panel (e.g., AUC 0.91–0.76 vs 0.96–0.96; accuracy 0.87–0.75 vs 0.87–0.81; 95% CIs in Supplementary Table S2, Supplementary Digital Content 1, http://links.lww.com/CTG/B394). This suggests that the larger panel carries complementary information that stabilizes generalization. Consistent with this, correlation analysis showed coordinated signals (H4.16-ANXA2; DEFA1B-TUFM-CEACAM7; moderate coregulation of PLA2G1B with ANXA2), and SHAP indicated small but consistent contributions from proteins such as zinc finger protein 790 despite weak univariate signal. Given the proof-of-concept scope and small cohort, we therefore retained all 16 proteins to avoid discarding useful biology and to limit overfitting to a narrowly defined subset. Practical downselection will occur in next step research during targeted multiple-reaction monitoring verification, guided by analytical criteria (unique peptides, transition quality, matrix effects, linear range/lower limit of quantification, and repeatability) and by performance in larger, multicenter cohorts.

A key limitation of this study is the small cohort size, including only Canadian patients with IBD aged 18 and older, which may limit its generalizability. The data set size is also relatively small for building a fully functional predictive machine learning model with 16 features. Although this study serves as a proof of concept to demonstrate that stool protein biomarkers can classify CD and UC, a larger data set would undoubtedly improve the accuracy and stability of the model. In addition, the accuracy achieved based on the use of a single-center cohort, which may reflect site-specific patterns; future studies will incorporate samples from multiple centers to enhance model robustness and external validity.

Although batch effects were corrected, all samples were analyzed on a single mass spectrometer, potentially limiting reproducibility across laboratories. Validation on multiple spectrometers would improve applicability. In addition, as diagnoses were based on clinical history, endoscopic, and histopathological findings, a small risk of misdiagnosis remains, emphasizing the need for larger, more diverse cohorts. Future efforts will focus on the clinical biomarker approach using targeted mass spectrometry.

In conclusion, this proof-of-concept study demonstrates the potential of SWATH-DIA proteomic profiling for distinguishing CD from UC using stool samples. A machine learning model built on 16 key protein biomarkers achieved an AUC of 0.96 in both training and blind test data sets, highlighting its reliability. ANXA2, CEACAM7, PLA2G1B, APCS, and TUFM consistently ranked as top features, supported by SHAP, correlation, and network analyses. Introducing this noninvasive diagnostic method lays a strong foundation for reducing reliance on invasive procedures and improving the quality of life for individuals with IBD.

To translate these findings toward clinical use, we will develop a targeted multiple reaction monitoring assay for the 16 identified proteins, followed by downselection to a compact panel based on peptide and transition quality, linearity and lower limit of quantification, reproducibility, and cost-effectiveness. This optimized MS-based panel will then be validated in multicenter cohorts with a larger number of samples to ensure robustness, generalizability, and performance across diverse clinical settings. Beyond CD-UC subtyping, the same workflow could be extended to tasks such as distinguishing IBD from non-IBD conditions or predicting disease activity.

## CONFLICTS OF INTEREST

**Guarantor of the article:** Jean-François Beaulieu, PhD.

**Specific author contributions:** E.S., D.G., and J.-F.B.: conceptualization. E.S., D.G., P.R., J.-F.N., and M.B.: methodology. E.S., D.G., M.M., P.R., and M.D.: collection of samples. E.S. and D.G.: data curation and formal analysis. E.S., F.B., and M.B.: statistical and machine learning analysis. E.S., D.G., M.D., M.B., F.-M.B., and J.-F.B.: funding acquisition. H.G. and J.-F.B.: resources. E.S.: writing—original draft preparation. E.S., D.G., F.B., M.M., P.R., J.-F.N., H.G., M.B., M.D., F.-M.B., and J.-F.B.: writing—review and editing. M.B, M.D., F.-M.B., and J.-F.B.: supervision. All authors have read and agreed to the published version of the manuscript.

**Financial support:** This work was supported by grants from Crohn's and Colitis Canada (grant 1031647) to M.D., M.B., F.-M.B., and J.-F.B. and the Natural Sciences and Engineering Research Council of Canada through a cooperative ENGAGE grant to J.-F.B. with Allumiqs Solutions. E.S. was the recipient of a doctoral studentship from the Faculty of Medicine and Health Science of the Université de Sherbrooke. D.G. was the recipient of an MITACS postdoctoral fellowship obtained in collaboration with Allumiqs Solutions. J.-F.B. was the recipient of the Canada Research Chair in Intestinal Physiopathology. The funders had no role in the design of the study; in the collection, analyses or interpretation of the data; in the writing of the manuscript; or in the decision to publish the results.

**Potential competing interests:** H.G., J.-F.N., and D.G. are the CSO & Director and employees, respectively, of Allumiqs. E.S, D.G., M.M., H.G., J.-F.N., and J.-F.B. are the inventors of the intellectual property owned by TransferTech Sherbrooke, a valorization society for the Université de Sherbrooke, and the subject of a provisional patent. The other authors declare no conflict of interest.

**Ethical considerations:** The research protocol for accessing stool samples from patients who have been tested for f-cal included a reverse consent procedure for using residual stool samples and accessing the related clinical data on the Ariane network for diagnosis. The study was conducted in accordance with the Declaration of Helsinki and approved by the Institutional Research Ethics Committee of the CIUSSS de l'Estrie—CHUS (protocol code 1991-17, 90-18, last date of approval 2025-08-27).

**Disclosure of AI tool use:** ChatGPT (OpenAI) was used to assist in clarifying and/or simplifying portions of the manuscript text. The authors reviewed and edited all content to ensure accuracy and adherence to publication standards. The authors take full responsibility for the final content of the manuscript.

**Data availability:** The mass spectrometry proteomics data have been deposited in the ProteomeXchange Consortium through the PRIDE partner repository http://www.ebi.ac.uk/pride with the data set identifier PXD057120.

**Study Highlights**

**WHAT IS KNOWN**

✓ A reliable noninvasive method for differentiating Crohn's disease (CD) and ulcerative colitis (UC) is lacking.
✓ Current diagnosis relies on invasive colonoscopy and histopathology.
✓ Fecal proteins reflect intestinal inflammation, making them potential biomarkers.
✓ Existing proteomic studies have not provided a consistent model for distinguishing CD from UC.

**WHAT IS NEW HERE**

✓ High-throughput sequential window acquisition of all theoretical mass spectra mass spectrometry and machine learning successfully classified CD and UC using stool biomarkers.
✓ A predictive model based on 16 proteins achieved area under the curve 0.96 in both training and test data sets.
✓ Annexin A2; carcinoembryonic antigen-related cell adhesion molecule 7; group IB phospholipase A2; amyloid P component, serum; and TUFM emerged as the most significant biomarkers.
✓ Developing a noninvasive stool test for CD-UC differentiation improves clinical management.

## Supplementary Material

## ABBREVIATIONS:

aCD: active Crohn’s disease
aUC: active ulcerative colitis
AUC: area under the curve
CD: Crohn’s disease
DEPs: differentially expressed proteins
DIA: data-independent acquisition
DP: detected proteome
GO: gene ontology
IBD: inflammatory bowel disease
KNN: K-nearest neighbors
LC-MS/MS: liquid chromatography-tandem mass spectrometry
NB: Naive Bayes
PCA: principal component analysis
RF: Random Forest
RFE: recursive feature elimination
ROC: receiver operating characteristic
RRF: regularized Random Forest
SHAP: SHapley Additive exPlanations
SVM: Support Vector Machine
SWATH: sequential window acquisition of all theoretical mass spectra
UC: ulcerative colitis
WP: whole proteome
XGBoost: eXtreme Gradient Boosting
