# Supplementary material for: Stool-Based Proteomic Signature for the Noninvasive Classification of Crohn's Disease and Ulcerative Colitis Using Machine Learning
Source: Clin Transl Gastroenterol. 2025 Oct 2;16(11):e00925. doi: 10.14309/ctg.0000000000000925 (PMC12637349; doi:10.14309/ctg.0000000000000925)
Supplement: Supplementary file 1 [file ct9-16-e00925-s001.docx]

**Table S1**. Network centrality and betweenness values for DEPs in the protein interaction network. Selected 16 proteins are highlighted in gray.

| Proteins | Centrality | Betweenness |
| --- | --- | --- |
| LYZ | 0.3529 | 0.3076 |
| GAPDH | 0.3235 | 0.2789 |
| LCN2 | 0.2353 | 0.1433 |
| ACE | 0.2353 | 0.1075 |
| PLG | 0.2059 | 0.0402 |
| AMY2B | 0.1765 | 0.1231 |
| AMY2A | 0.1765 | 0.1231 |
| DPP4 | 0.1765 | 0.1076 |
| S100A9 | 0.1765 | 0.0492 |
| CPA1 | 0.1471 | 0.1658 |
| H3-3B | 0.1471 | 0.0556 |
| APCS | 0.1471 | 0.0517 |
| PRTN3 | 0.1471 | 0.0345 |
| PIGR | 0.1471 | 0.0163 |
| SERPINA3 | 0.1471 | 0.0137 |
| AZU1 | 0.1471 | 0.0122 |
| SERPINC1 | 0.1471 | 0.0105 |
| OLFM4 | 0.1176 | 0.2139 |
| RPS27A | 0.1176 | 0.0174 |
| DEFA1B | 0.1176 | 0.0052 |
| CLCA1 | 0.0882 | 0.1658 |
| SLC26A3 | 0.0882 | 0.0285 |
| CLCA4 | 0.0882 | 0.0285 |
| HBB | 0.0882 | 0.0049 |
| CELA3B | 0.0882 | 0 |
| CPA2 | 0.0882 | 0 |
| PLA2G1B | 0.0882 | 0 |
| JCHAIN | 0.0588 | 0 |
| TUFM | 0.0588 | 0 |
| ANXA2 | 0.0588 | 0 |
| H4-16 | 0.0588 | 0 |
| CEACAM7 | 0.0588 | 0 |
| DMBT1 | 0.0588 | 0 |
| SOD2 | 0.0588 | 0 |
| MEP1A | 0.0294 | 0 |

**Table S2**: Performance of the 16-protein discovery panel vs a 5-protein core (ANXA2, CEACAM7, PLA2G1B, APCS, TUFM) for CD vs UC classification using Naive Bayes. Models were trained with repeated 10-fold CV×3 an d SMOTE on the training cohort and evaluated on an internal prospective test cohort. Accuracy is reported with 95% Wilson CIs; AUC with 95% DeLong CIs. Best hyperparameters are shown at right. The 5-protein model retained good discrimination but showed a larger train–test gap (e.g., AUC 0.91→0.76) than the 16-protein panel (AUC 0.96→0.96), consistent with mild overfitting and/or loss of complementary signal.

|  | Performance | Train | Test | Hyperparameter |
| --- | --- | --- | --- | --- |
| 16 Biomarkers | Sensitivity | 0.86 | 0.81 | fL=0.25 usekernel= FALSE adjust=3 |
|  | Specificity | 0.89 | 0.80 |  |
|  | Precision | 0.90 | 0.90 |  |
|  | F1 score | 0.86 | 0.86 |  |
|  | Accuracy | 0.87 (0.75-0.94) | 0.81 (0.54-0.95) |  |
|  | AUC | 0.96 (0.91-1.00) | 0.96 (0.88-1.00) |  |
| 5 Biomarkers | Sensitivity | 0.86 | 0.73 | fL=0.75 usekernel= FALSE adjust=0 |
|  | Specificity | 0.89 | 0.80 |  |
|  | Precision | 0.93 | 0.89 |  |
|  | F1 score | 0.89 | 0.80 |  |
|  | Accuracy | 0.87 (0.75-0.94) | 0.75 (0.49-0.93) |  |
|  | AUC | 0.91 (0.82-0.99) | 0.76 (0.49-1) |  |
